# Practical Tips for Successful Implementation of Educational Innovations: Project Management Tools for Health Professional Educators

**DOI:** 10.15694/mep.2018.0000105.1

**Published:** 2018-05-22

**Authors:** Elisabeth Frieda M. Schlegel, Kevin Brian D. McLeod, Nancy J. Selfridge

**Affiliations:** 1 Donald and Barbara Zucker School of Medicine at Hofstra/Northwell; 2 AdTalem Global Education; 3 Ross University School of Medicine

**Keywords:** Implementation of educational programs, project management, project template, project scope, problem-solving

## Abstract

This article was migrated. The article was marked as recommended.

**Background:** Growing demands from health professional educators lead to expectations for smooth implementation of high-stake endeavors such as developing and successfully launching educational innovations ranging from curricular sessions to new programs under time constraints. Usually, multiple requirements, resources, and processes need to be identified, evaluated, and effectively utilized, making the process difficult and confusing. Personnel and operations must be aligned to create momentum for a successful workflow, which requires planning and negotiation with stakeholders. Increasingly, project management strategies and skills have been recommended to medical educators for accomplishing these goals; however, to-date no practical tools or templates have been provided to support and guide educational implementation processes in a practical fashion.

**Aim:** Adjusted to the needs of medical educators across all healthcare professions, we provide interactive templates and tools in the appendix, which walk readers through the implementation of a complete educational project from start to finish.

**Methods:** Using project management guidelines established by the Project Management Institute (PMI), the global credentialing body for the project management profession, the tools and templates follow best practices that were aligned with standards of curriculum development (six-step approach) as published by Kern and colleagues ( Thomas, Kern, Hughes & Chen (Eds.), 2016).

**Results:** We identified a practical workflow for implementing educational innovations and developed interactive tools and templates as guides for tracking and presenting progress for the launch of educational projects of any scale, large or small.

**Conclusions/summary statement:** Developing and implementing complex, multistep endeavors such as collaborative educational sessions or programs can be mastered successfully using project management tools and templates. Based on international project management guidelines and best practices, the authors outline how project management tools and templates allow medical educators to streamline successful systematic planning, engage in creative-problem solving and establish consensus under time constraints.

## Introduction

Successful implementation of educational programs for health professional educators requires leadership and management skills, overseeing coordinated schedule-intensive operations and leading teams composed of diverse professions and resources ( Doherty, 2010; Gorsky, Mann & Sargeant, 2016; Sipes, 2016; Thomas, Kern, Hughes & Chen (Eds.), 2016). Effective communication across academic and operational departments is key to completing curricular projects on time. Using a systematic approach to coordinate, inform, and guide development for projects of any scale - large or small - is fundamental for success ( Rizzo, 2016).

As defined by the Project Management Institute (PMI), the global credentialing body for the project management profession, a project is “a temporary endeavor undertaken to create a unique product, service, or result” ( PMI, 2017, p. 542). A project has distinct starting and ending points and, at a minimum, a schedule, a budget (equivalent to worktime in academia), a scope and resources. Project management, the application of supportive knowledge, skills, tools, and techniques, leads to accomplishing educational projects in a step-by-step fashion. The organizing health professional educator who assumes the role of project manager acts as point of contact to ensure timely completion of the project through collaborative team efforts.

With the growing demand to plan, oversee the development of, and implement new curricula and programs, educational developers have been encouraged to take on the role of project managers ( Thomas et al., 2016). Moreover, the application of the project management process on implementation of educational innovations has been recognized for decades (Ball & Cummings, 1973). In addition, faculty and staff development efforts reflect the new obligations and roles of health professional educators to include management and leadership skills that surpass the traditional roles of academic teachers ( Mennin, Kalishman, Eklund, Friedman, Morahan & Burdick, 2013; Rizzo, 2016). It has been recognized that project management structures promote implementation of curricula and technology innovations, increase productivity and reduce work stress and interpersonal conflict due to shared responsibility for completing projects. Planning templates and tools drive the process and help to keep everybody on task (Ball & Cummings, 1973; Loo, 2003; Rizzo, 2013; Thomas et al., 2016).

In this article we provide interactive project management tools and templates based on the most common needs of medical educators; furthermore, we present 12 tips aligned with 12 interactive templates. These are combined into a downloadable Microsoft (MS) Excel booklet that walks the educator from planning to launch of a program. Each template can be accessed by clicking on the tab at the bottom. It is important to notice that the complexity of the project dictates the amount of planning and preparation required. Smaller projects might be successful using only the inventory checklist (Tip 2) and a matrix schedule (Tip 6) after clarifying the scope (Tip 1), while larger projects require more documentation for managing the deliverables. The reader can pick and choose. The tips and associated templates follow the major project management processes, provide tools aligned with medical education and are adjusted to stages in planning of educational programs. All templates are customizable in MS Excel and can be printed to the Adobe Portable Document Format (pdf).

## Tip 1

### Establish the Scope of an Educational Project

The scope (Tip 1) describes the planning timeframe, a description, learners’ goals and objectives, and educational strategies needed to deliver the content, as well as outcome measures, which align with Kern’s six-steps approach of program development ( Thomas et al., 2016). Generating the scope is a collaborative effort, guided by the lead educator in the role of the project manager, together with collaborating faculty, staff, and experts comprising the project team.

The project scope also draws the boundaries of the project, documenting what is implemented and what is not. As an example, in the context of educational information technology (IT), interested users from other departments might suggest technology functionalities necessitating additional project objectives. While scopes evolve over the life cycle of the project, due to feedback designed to refine objectives ( Mennin et al., 2013), boundaries of the scope need to be negotiated with an awareness of the collegial nature of academia in order to stay within the set timeframe.

**Figure:**
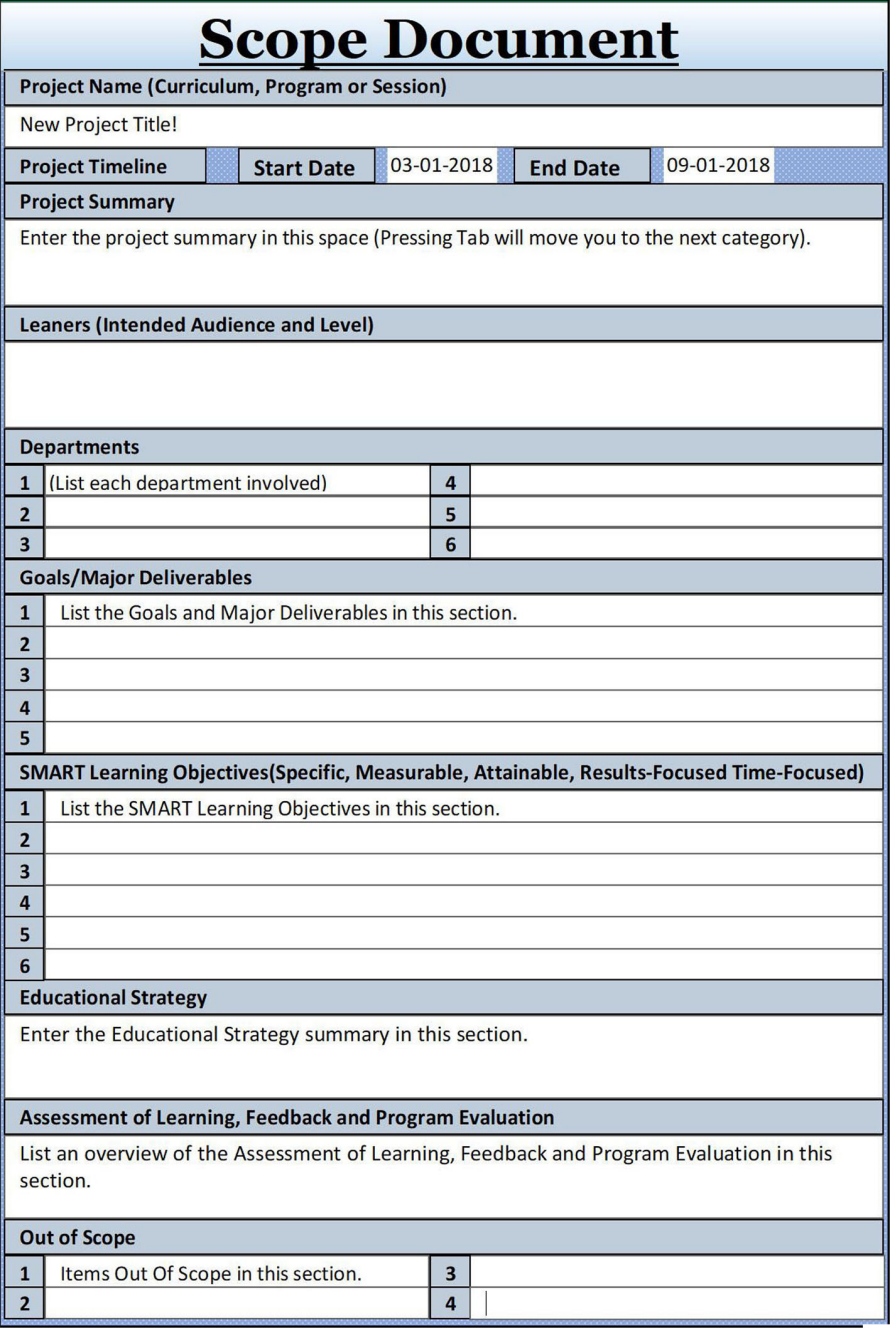


## Tip 2

## Identify and Secure Resources and Support through an Inventory Checklist

Identifying and securing resources and key personnel is needed to implement an educational project successfully ( Thomas et al., 2016; Gorsky et al., 2016). The lead educator as project manager initiates meetings with the immediate collaborators, who become the project team; they then identify sufficient human and material resources, political buy-in and administrative structures. To answer the who/what/when/where/why/how-much/how-long questions, the inventory checklist supports the process of compiling resources, listing items such as personnel, time, facilities, tools, technology, and cost. This checklist will also quickly relate to the feasibility of the project.

Securing stakeholder support and political buy-in from leadership is a make-or-break component, ensuring that leadership is aware and in favor of the educational project being planned and implemented ( Mennin et al., 2013). Documenting internal and external approvals of stakeholders - from students to academic leadership and communities - is necessary for success. These approvals must be evidenced in written communication.

**Figure:**
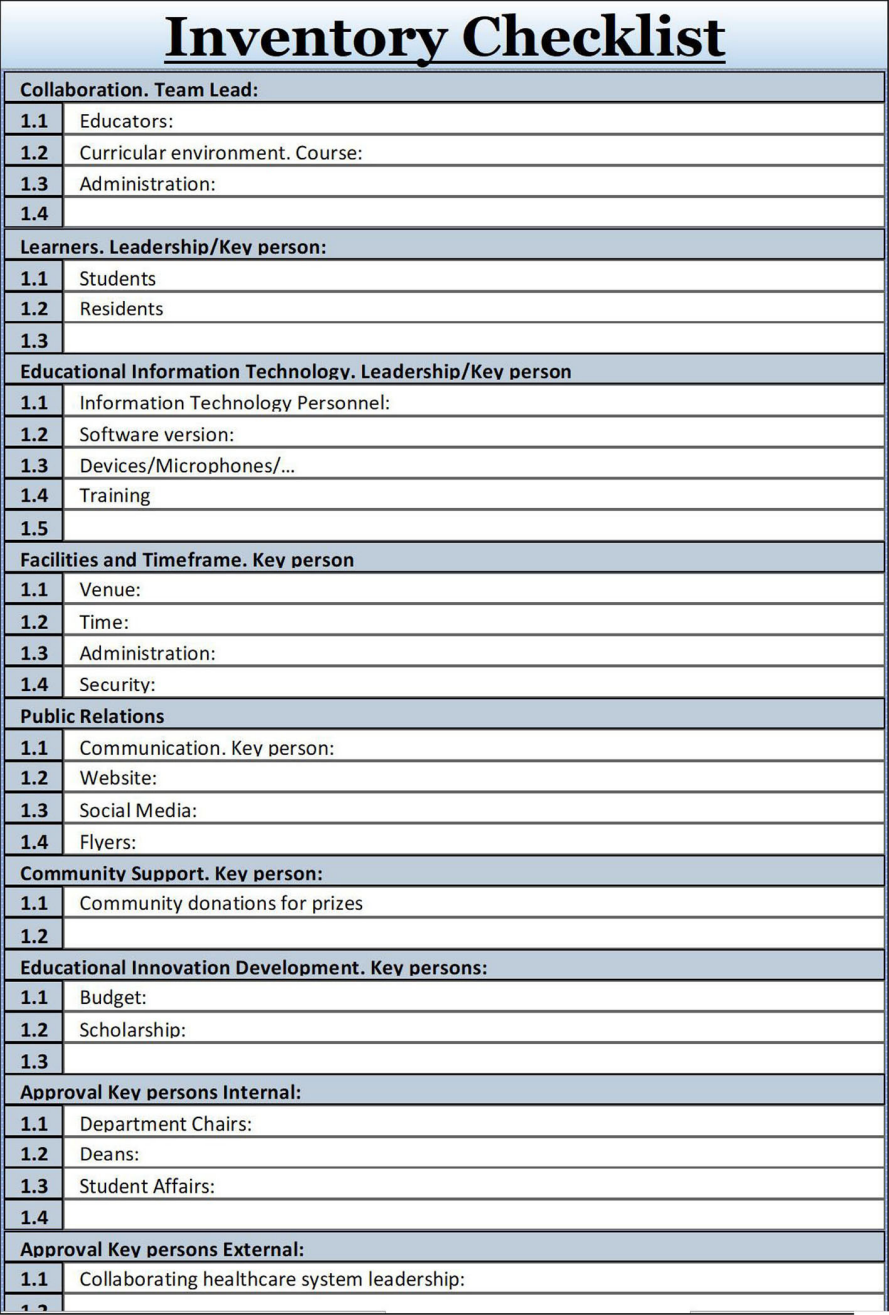


## Tip 3

## Determine Manageable Tasks through the Work Breakdown Structure

Project planning has been recognized as promoting effective work habits. It consists of efforts established in the scope to fine-tune and define the objectives before developing the course of action required to reach these objectives ( Loo, 2003). At this point, project managers and teams have to look more closely into the instructional design and educational strategies, breaking down the project into multiple tasks or steps that proceed in a parallel or sequential manner ( Williams van Rooij, 2011; Pan, 2012). Also, workload demands are estimated and might require adjustment of the objectives. As an example, if the curriculum goal is the generation of online modules, an expert author must deliver content to the web application architect first for uploading.

The work breakdown structure (WBS) is used for defining and managing the project scope. It is a commonly used tool for brainstorming and it visibly shows how project deliverables and objectives are broken down into work packages or tasks. The visualization reportedly provides clarity of the overall endeavor and team confidence ( Rizzo, 2016). Completing the WBS includes dividing the project into manageable first-order main tasks. A hierarchical breakdown flow of lower-order work packages, called subtasks, demonstrates the sequence of the project work, easing construction of a timeline, and allowing deliverables to be planned, monitored, and controlled. As an option, the tasks can be copied forward into the overall big-picture schedule developed in Task 6. The order of the tasks will be assigned next.

## Tip 4

### Determine the Sequence of Tasks

Putting all the steps in an order provides another level of clarity. Sequencing activities is the process of identifying and documenting interrelationships among the project activities for developing the project schedule. The key benefit is defining the logical sequence of work to obtain a smooth workflow. It also provides an idea of dependencies about which task needs to be finalized before another one can be started, tasks that can be carried out concurrently and critical tasks that need to happen for the project to move forward and meet the deadline. As an example, for online modules to be implemented, the upload of content by the application specialist cannot start until the content is finalized by the content expert. On the other hand, enrollment of students in the course and uploading of content are independent of each other and can happen at the same time.

The sequence chart aligned with Tip 4 allows determination of the start and end date of each task documented in the previous work breakdown structure. Each task from the WBS is assigned a number in the sequence, a start and an end date, resulting in a logical flow moving forward. Arrows allow moving the tasks and all aligned subtasks to a different place in the sequence. Selected tasks can also be marked as milestones for determining the accomplishment of those tasks on critical dates.

**Figure:**
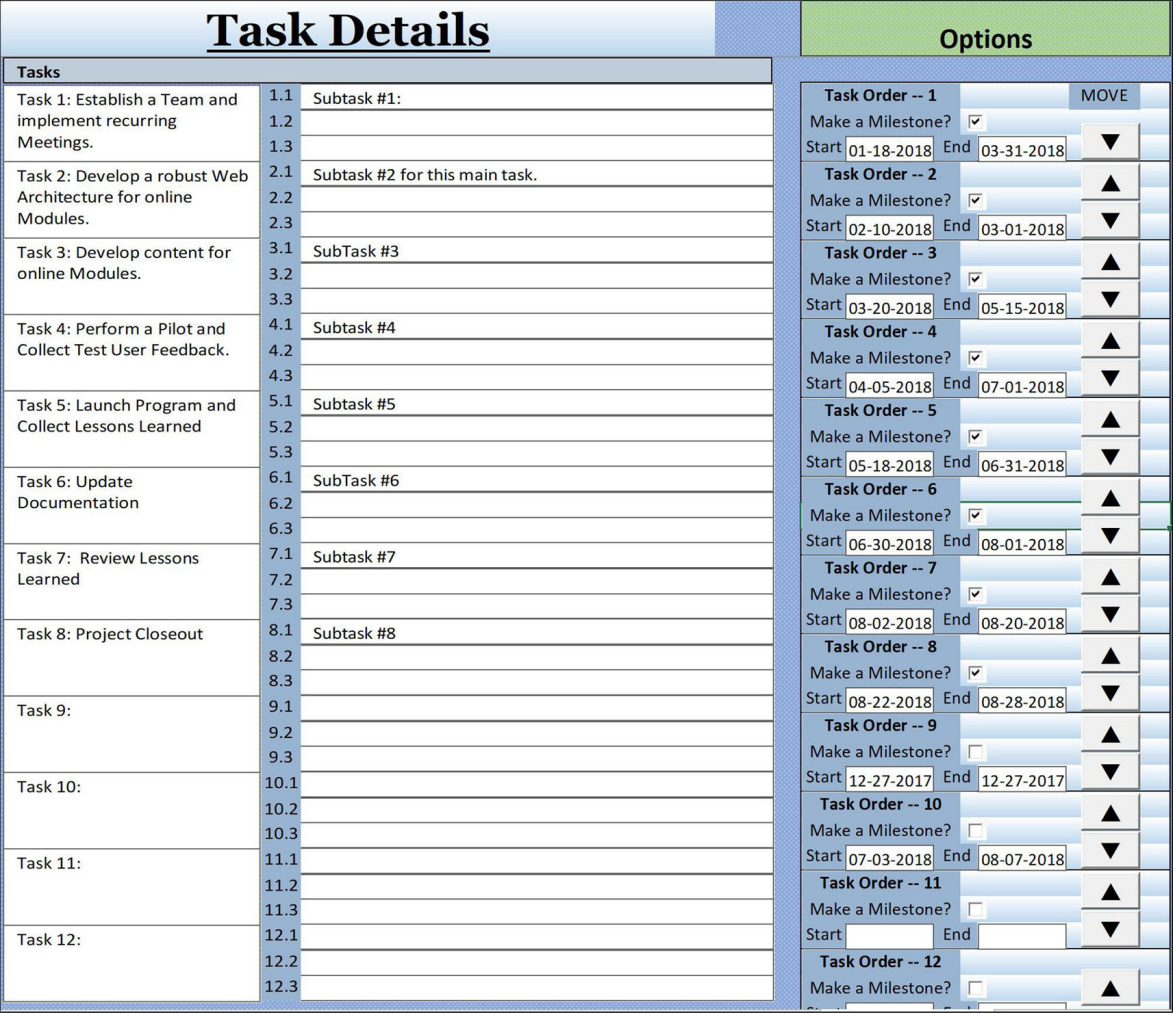


## Tip 5

### Identify Significant Dates and Establish Milestones

One feature of project management is the special attention to scheduling ( Heagney, 2016). The primary goal is meeting the final deadline, which is also the launch of the educational program being planned, or the first session with your learners. This date is, in project management jargon, a “critical” date, which must be achieved at a certain time.

For ease of managing dates in a busy academic environment, a graphical representation of selected tasks associated with significant dates and milestones will be established in the template termed Tip 5 - Milestones. A milestone represents a point in time with specific importance to the project, such as finishing the development of an activity ready to be applied with the learners or scheduling a pilot test of a program. Assigning milestones to the educational project allows breaking of the project into major portions each composed of several tasks divided into subtasks. Upon marking the completion of important tasks in Tip Sheet 4, all milestones can be viewed in chronological order in Tip Sheet 5. Please note: Projects have only a few milestones that can be achieved and then celebrated, if completed on time. The milestone representation in Tip Sheet 5 ensures that crucial work is tracked in a timely fashion.

**Figure:**
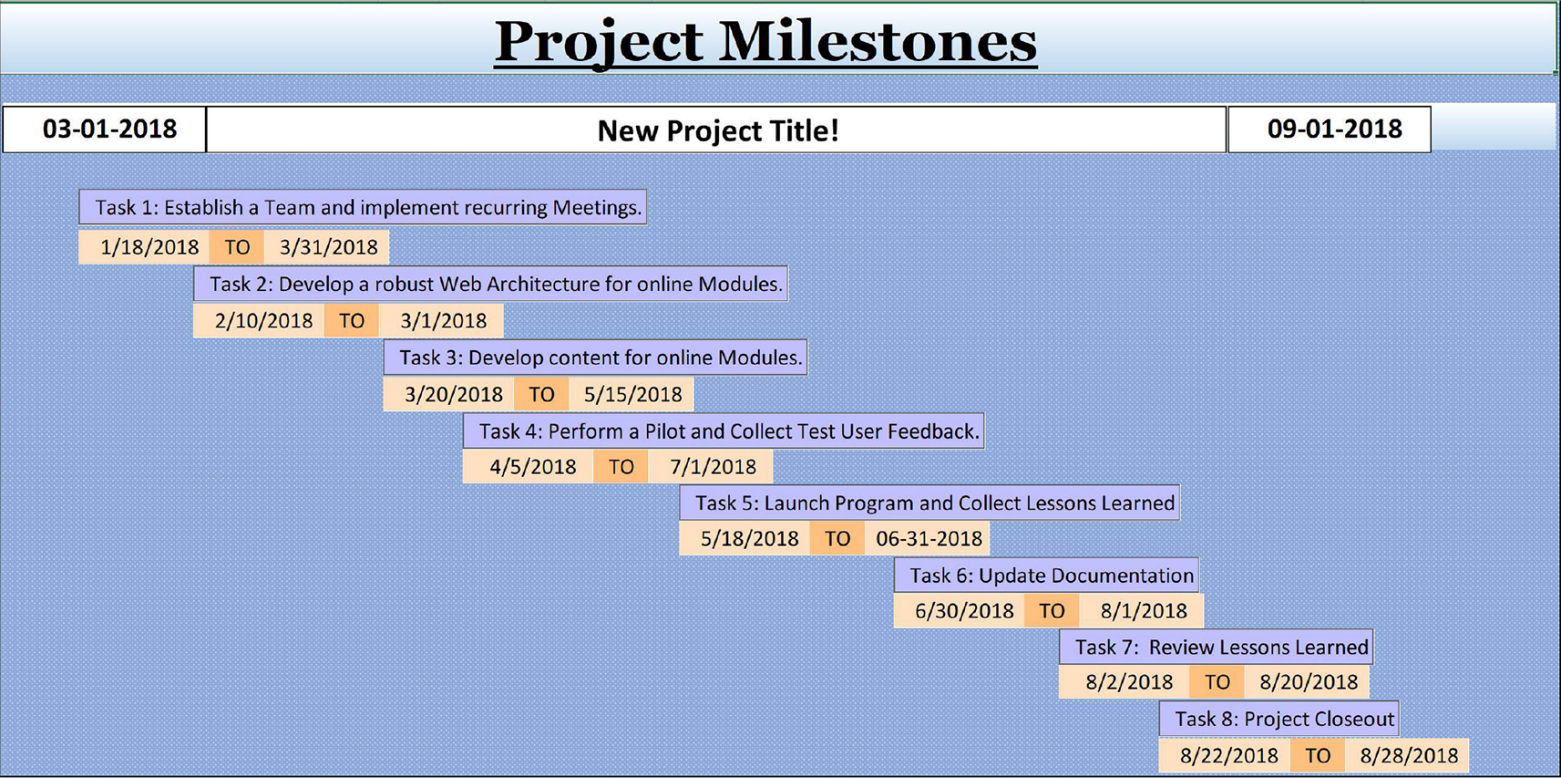


## Tip 6

### Align Resources with the Schedule

By combining tasks, timeframes and milestones with leadership, a matrix-type schedule is accomplished (Tip Sheet 6). Entries from Tip 3 are initially copied forward by choosing the button “Update Tip 6 Matrix.” This matrix provides the entire project framework, can easily be revised, and is suitable for sharing through commonly accessible platforms. Leadership, key initiative team members, assessment methods, performance indicators and outcomes can now be documented, providing a visual representation for the educational project leader and the team, supporting communication and progress reviews. Key initiative team members - experts with a specific skill such as IT staff or instructional designers - are added to clarify assigned responsibilities to accomplish or guide completion of a specific task and will vary from task to task.

The field Assessment Method/Performance Indicator helps in achieving the desired quality of a specific task, including non-negotiables or must-haves, such as the use of a document style required by an institution. The desired outcomes of tasks and subtasks must be clearly described as the desired end results. As an example, the outcome for the high-level task “develop a storyboard for a modular online course” might be “sequence of publishable web pages ready to be populated with content.” The outcome for the subtask “develop the landing page for a modular online course” might be “placement of a representative image, title and subtitle, menu, and contacts”. Completing the fields as a team ensures collaborative solutions and buy-in. Also, the matrix is suitable for providing a status update to stakeholders such as approving bodies. The document is further valuable for maintenance and improvement of the educational project once established, especially for running educational sessions that occur infrequently, such as major educational games or interprofessional case sessions requiring the management of participants from different healthcare disciplines. Some fields have been prefilled with a fictitious modular online program as examples.

## Tip 7

### Manage and Involve Your Stakeholders

Stakeholders are individuals, groups, or communities, such as the home institution or departments, who have an interest and a role in the project, as well as expectations for the purpose, goals and intended results of the educational intervention or program ( Larson & Lockee, 2014; Russell, 2015). Stakeholders may be comprised of, for example, students, the medical education project team, functional managers and administration, as well as organizational groups, such as academic societies outside the organization. Stakeholders have different functions and exert various degrees of influence on the success of a project, so involving them early in the project management process to manage their expectations is key. Strategic working sessions with key stakeholders to promote successful implementation of an education session is recommended ( Thomas et. al., 2016).

The stakeholder directory contains stakeholders and their functions in the project, thus helping to manage stakeholders’ degrees of support and collaboration through providing communication strategies for consensus (see Tip 8), but also risk management (see Tip 9). For example, the sole technician setting up educational software has a stakeholder key role, which might even necessitate cross-training back-up individuals as preventive and supportive measures. The dean of the university, on the other hand, has great influence over any educational project but might not be closely involved or expect routine reports through committees.

The stakeholder directory lists stakeholders and their functions in the project depending on their degree of influence and involvement. It not only provides an overview of responsibilities toward stakeholders, but it also displays stakeholder expectations and keeps stakeholder relationships vibrant. At the top of the directory list are the stakeholders who exert considerable influence, have great interest in the project and need to be kept informed about the progress. Depending on the organizational environment of the project, this level might contain institutional leadership, but also sponsors, or supporting senior faculty colleagues. At the bottom of the directory list are stakeholders who have less involvement and might only receive updates as needed, such as standing university committees.

## Tip 8

### Plan the Communication: Sharing Decisions and Credit of Successes

Great communication includes sending regular status reports and project updates. Smaller projects might use regular email notifications; more complex projects need a detailed communication plan defining information requirements plus outlining the appropriate communication technology. Effective communication includes developing a list of the stakeholders or designees represented in Tip Sheet 7, followed by adding communication methods for project information in Tip Sheet 8. Entries are copied forward.

Preferred ways of communication with stakeholders need to be established at the beginning of the project and stakeholder consultation through meetings or separate consultation depends on the individual role and degree of influence. Stakeholder expectations can be streamlined at the beginning of a project through personal communication, surveys or interviews.

Tip Sheet 8 contains the basics for consensus and buy-in with important individuals. Each person or her/his designee included in the stakeholder directory should receive regular status reports. Keeping in mind people’s different communication styles, several ways of contacting a stakeholder should be included.

**Figure:**
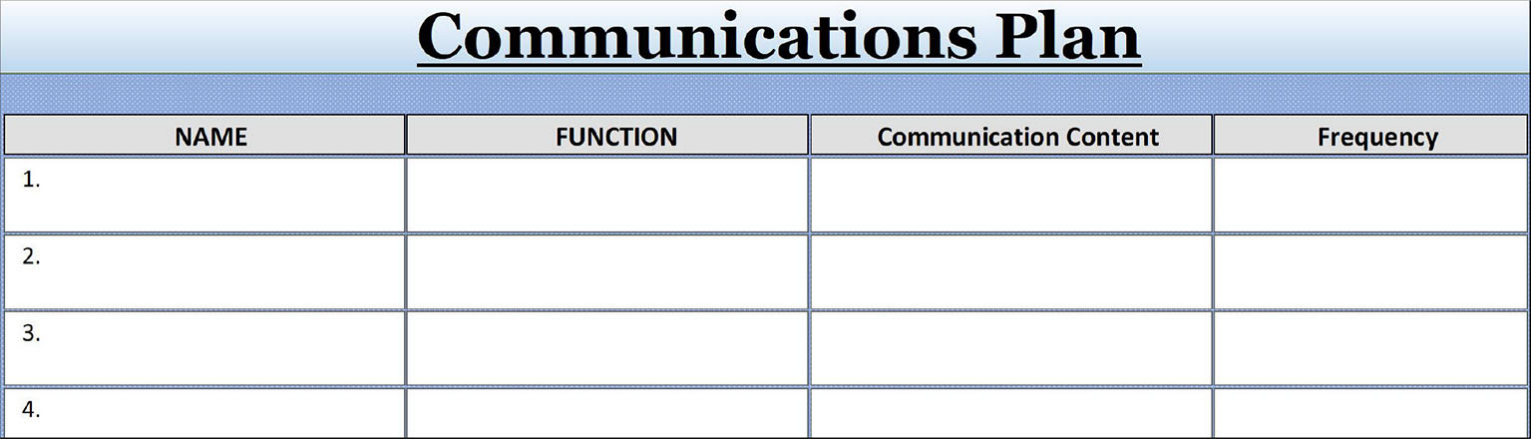


## Tip 9

### Foresee and Disarm Challenges through Managing Risks

Challenges are part of a project and can be dealt with through anticipating and leveraging risks ( Mennin et al., 2013). Risks encountered during a project might include, for example, a short-notice upgrade of the essential teaching software, budget cuts, exit of a crucial team member, or sudden loss of a classroom needed for group sessions due to inclement weather. Analyzing risks and managing them upfront allows an educational project manager to anticipate situations or glitches and develop contingency plans, making additional time for status meetings for updating and managing expectations. Tip Sheet 9, the risk plan, allows listing the anticipated risks together with its level of impact (low/medium/high) next to the strategy about how to mitigate it. This straightforward risk-scenario planning table keeps the project leader prepared and provides a preventive response - a plan B to keep the project on track.

**Figure:**
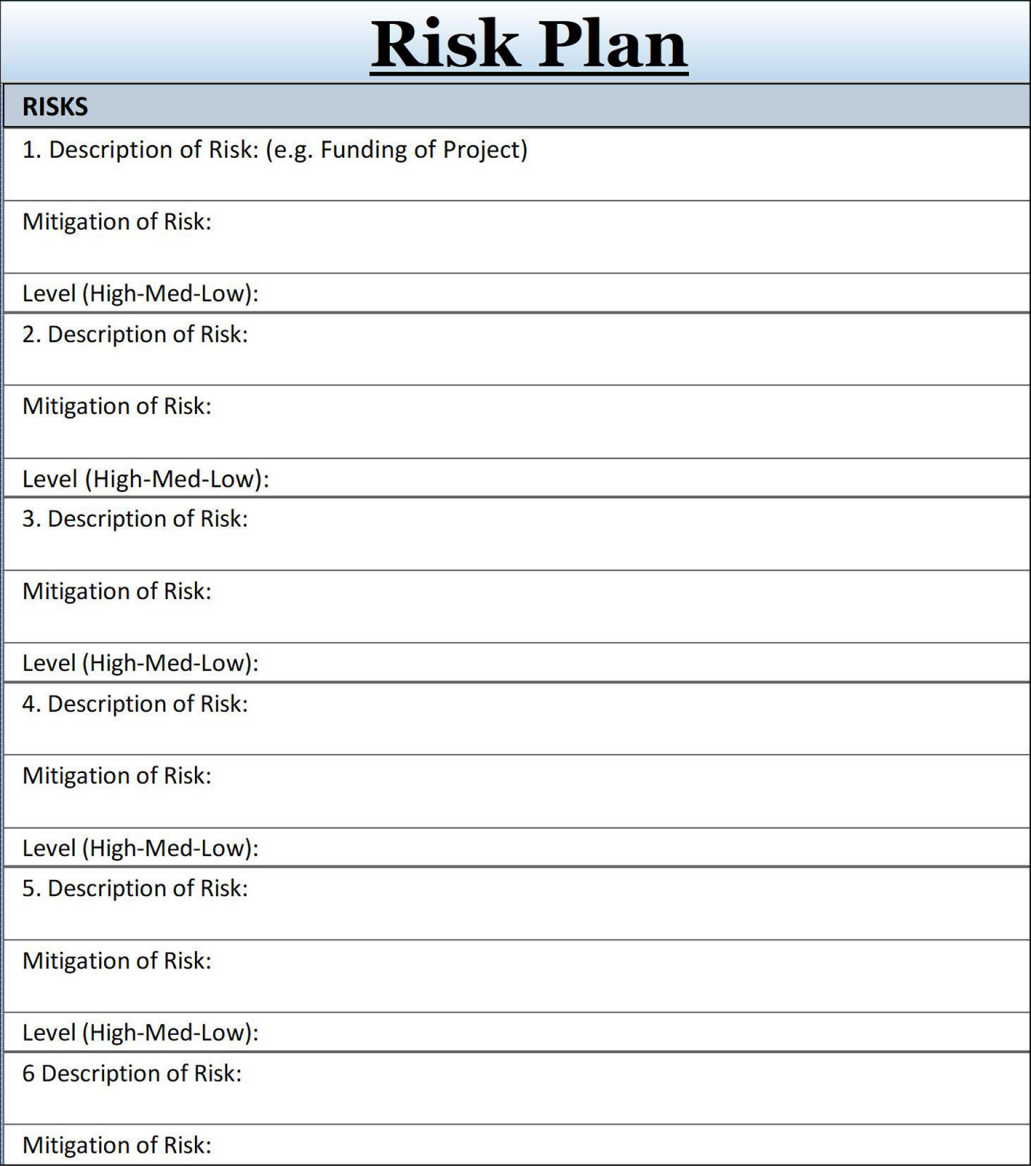


## Tip 10

### Keep the Project Quality High: Integration of Communication and Management

The main principle of project quality management is ensuring the project will meet or exceed stakeholders’ needs and expectations. The project team must develop a good relationship with key stakeholders, especially the learners, who are the beneficiaries of the project. A consensus must be established about what quality means, which oftentimes equals institutional objectives being met or educational software delivering content smoothly. Indicators for desired quality can be documented early in the planning stages in Tip Sheet 6 (Task Matrix) in the column Assessment Method/Performance Indicator.

Indicators for quality and success must be associated with metrics to measure quality. As mentioned by Mennin and colleagues (2013), a great way to measure the quality of an educational project is conducting a pilot study of a prototype and collecting comprehensive feedback through, for eaxmple, surveys exploring indicators of effectiveness, such as instructional design or the functionality of instructional technology ( Lam, Hung, Chan, Yan, & Woo, 2011). A less complex solution to quality measurement is a dry run of a portion of the session with note taking or use of a pre-determined checklist. Feedback will be implemented immediately after completing the pilot to be ready for the launch date, which might draw attention from leadership, learners and the institutional community alike.

**Figure:**
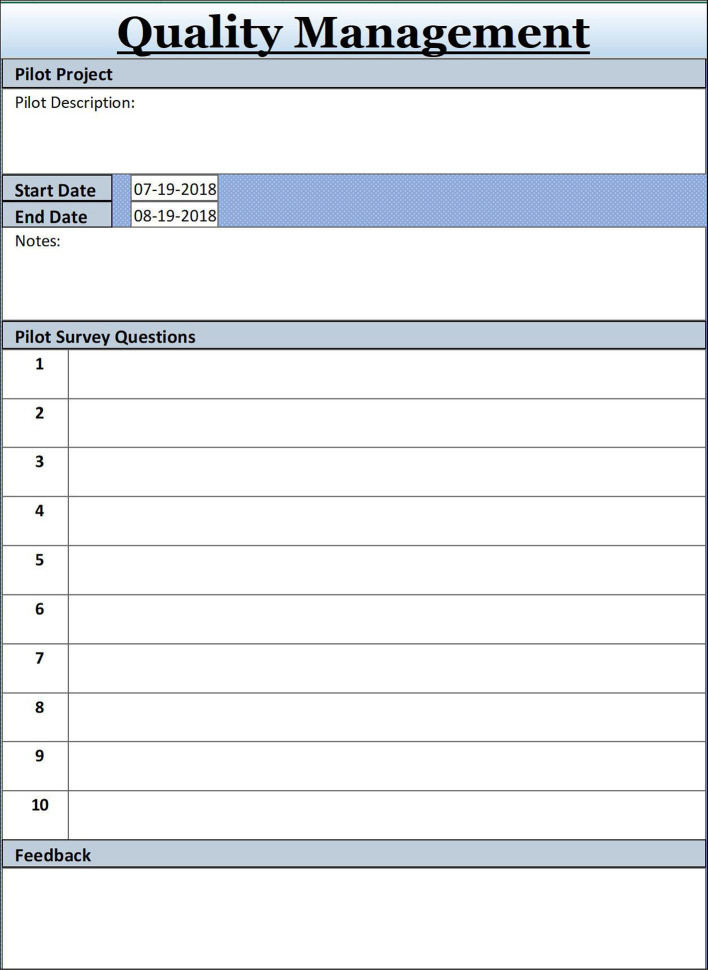


## Tip 11

### Launch the Project: The Big Day of Deployment!

The big day has finally arrived: today is the start of the program. Comparable to a countdown, project milestones and the scope have been accomplished, feedback has been implemented and content has been proofread - everything is ready.

A few items in place prior to deployment will ensure a smooth launch of the program or session, termed the deployment plan. This plan can be created ahead of time through completion of Tip Sheet 11 (Deployment Plan). As deployment is rolled out, each executed task can be checked off. The following might aid the big day.

In addition to establishing social media and websites, announcements and promotional material (flyers) should be sent out to stakeholders and the community at least one month, one week, and one day prior to the launch. Arrangements for a photographer or video recorder compliant with institutional policies will ensure documentation for follow-up communication, research, and presentations. Arranging for on-site support is highly recommended. This may include IT staff ready to solve an issue immediately, or a kind and attentive colleague attending the session for additional support if needed. Emergency phone numbers should be available in plain sight and saved on the phones. On the launch day, setting up early is advisable, providing microphones, water bottles and tissues. A notepad placed at the lectern helps when tracking unexpected situations. Smile and enjoy the day!

## Tip 12

### Reflect about Accomplishments and Collect Lessons Learned

The closing phase is the time and space for wrapping up the project, collecting lessons learned and inviting reflections about accomplishments ( PMI, 2017, p. 548). This is also the time for sending out the project close-out survey to stakeholders to collect feedback about the effectiveness of the project processes, general collaboration, resources allocation and overall satisfaction.

Lessons learned and captured along the way need to be discussed as a team and can be moved into the documentation of the course or session as historic items with recommendations for improvement. Archiving lessons learned together with the project documentation creates a prized repository of knowledge for future project success ( Loo, 2003; Doherty, 2010; Overgaard, 2010).

The meeting with stakeholders for reporting and closing will discuss the project close-out survey informing decisions and next steps, but also future directions; support for additional projects; best practices; and unexpectedly-produced products or software applications. Student assessment and institutional program evaluation data will probably not be available at the close-out meeting; rather, it will be discussed separately at the usual institutional level. These meetings are also the occasion for celebrations and thanking everyone involved for the success of the project.

## Conclusions

By supplying interactive project management templates, this article closes the gap between recommending and successful application of the use of project management strategies for implementing medical education innovations ( Gorsky et al., 2016; Sipes, 2016; Thomas et al., 2016). From planning flipped classroom design to incorporating active learning into a shared teaching session, growing demands from medical school faculty and other health professional educators, such as collaboratively implementing new programs or educational sessions, can be accomplished successfully with a systematic project management approach broken down into twelve tips. The many benefits for using project management guidelines include clear strategies, starting from defining the scope to establishing a network of collaborators and prudently navigating challenges ( Mennin et al., 2013). Additionally, this approach provides a big-picture view with exact progress tracking tools to keep busy medical educators up-to-date while they still carry out other responsibilities. It puts your mind at ease and allows you to keep it simple by choosing templates for specific processes.

Project management applications and tools allow collaborators to coordinate and communicate effectively, making use of the little time they have to get the job done.

## Disclosure statement

The authors report no conflicts of interest. The authors alone are responsible for the content and writing of this article.

## Take Home Messages

• Implementation of educational programs requires team efforts across academic and operational departments, and at times across different healthcare professions. Project management applications and tools allow collaborators to coordinate, communicate and proceed effectively under time constraints.
• We provide a series of interactive templates for monitoring progress and completion of educational projects for the health professions together with offering practical tips on how to use them.
• Aligned with applications and tools derived from the Project Management Institute (PMI), the global credentialing body for the project management profession, educational projects as multistep procedures can be completed in an organized fashion while keeping track of tasks, challenges, and accomplishments.

## Notes On Contributors

*Dr. Elisabeth Frieda M. Schlegel, MSc, PhD, MBA* is Associate Professor in the Department of Science Education at Donald and Barbara Zucker School of Medicine at Hofstra/Northwell.

*Kevin Brian D. McLeod, MET,* is business analyst in the Project Management Office of AdTalem Global Education.

*Dr. Nancy J. Selfridge BS, MD,* is Associate Professor and Chair of the Department of Clinical Medicine at Ross University School of Medicine.

## Appendices

Project Management Tools

The above link provides access to 12 interactive project management tools and templates based on the most common needs of medical educators. They are combined into a downloadable excel booklet that walks the educator from planning to launch of a program. Each template can be accessed by clicking on the tab at the bottom. Please note, the complexity of the project dictates the amount of planning and preparation required. Smaller projects might be successful using only the inventory checklist (Tip 2) and a matrix schedule (Tip 6) after clarifying the scope (Tip 1), while larger projects require more documentation for managing the deliverables - just pick and choose! All templates are customizable in MS Excel and can be printed to pdf.
