# Supplementary material for: Practical Tips for Successful Implementation of Educational Innovations: Project Management Tools for Health Professional Educators
Source: MedEdPublish (2016). 2018 May 22;7:105. [Version 1] doi: 10.15694/mep.2018.0000105.1 (PMC10699388; doi:10.15694/mep.2018.0000105.1)
Supplement: Peer review history

# Reviewer report: Reviewer response for version 1

**DOI:** 10.21956/mep.19564.r29014

**Published:** 2019-05-21

**Authors:** Ken Masters

**Affiliations:** 1 Sultan Qaboos University

This review has been migrated. The reviewer awarded 5 stars out of 5 The paper provides insight into the use of project management tools for implementing educational innovations. In addition to describing the set of tools in detail, the authors have provided a link them, in the form of spreadsheets.The paper and its associated spreadsheets form an outstanding piece of work. There are many project-management tools, but most are extremely difficult to use and/or are not free. This paper provides a set of useful, straight-forward and free tools to be used easily when implementing a new educational innovation. Although professional project managers may question the simplicity of the toolset, this is not aimed at professional project managers. If every medical educator had easy and direct access to a professional project manager, then such a paper and toolset would not be necessary. As that situation is unlikely to occur within the next century, and medical educators are routinely expected to initiate projects without any real training or guidance, this tool will provide invaluable scaffolding.My only regret is that I did not read this paper sooner – it would have served me well on a (not very successfully) recently-completed project.I have no hesitation in giving this paper the maximum score.

Reviewer Expertise:

NA

No decision status is available

# Reviewer report: Reviewer response for version 1

**DOI:** 10.21956/mep.19564.r29016

**Published:** 2018-12-17

**Authors:** Bonnie Taylor

**Affiliations:** 1 University of Texas Health Science Center - San Antonio

This review has been migrated. The reviewer awarded 3 stars out of 5 The authors have conscientiously contextualized project management as a crucial set of processes for curriculum development, as such I would deem this an important paper for readers who are a) new to the challenges of designing and developing a health professions curriculum. I believe health professions educators should definitely perform their due diligence before embarking on such an endeavor. My hesitation in lauding it as a fundamentally significant contribution in the field of health professions education stems from the general tone of offering “tips and tricks” that might be misconstrued as a suitable substitute for professional project management. Even the most dedicated organizing educator assuming the role of project manager should be advised at the outset to leverage a professional project management expert and to secure such a resource as early as possible, rather than relying solely upon a “do it yourself” approach to project completion. Numerous potential pitfalls await novices in any project management endeavor. In my experience as a professional trainer and educator in many sectors (including public, private, and not-for-profit organizations), I have found that the novice practitioner confronts several hazards, some of which might be implied within the tone and structure of this paper. As noted in another review, the set of tools looks promising, and can seem overwhelming. To be clear, my perspective includes the belief that project management-- like education in general and health education in particular—are actual professions meriting extensive study and practice, as are the fields of medicine, nursing, pharmacology, etc. Admittedly, one has to start somewhere. The authors’ effort to contribute a helpful resource is worthy of commendation and does contribute usefully to the domain of health professions educational development. I believe its strongest value is in being a rather concise curation of common tools used by project management professionals that could be referenced in parallel with consultation with an expert instructional design project manager steeped in the framework provided in the Project Management Body of Knowledge (PMBOK). Familiarity with this paper could support the development of a common lexicon for terms such as scope, risk, quality, and even project, none of which should be assumed to be understood by all stakeholders new to the challenges that lie ahead. For health professionals aspiring to professional educator status in the domain of project management, these practical tips serve as worthy advice to be heeded—with guidance.

Reviewer Expertise:

NA

No decision status is available

# Reviewer report: Reviewer response for version 1

**DOI:** 10.21956/mep.19564.r29017

**Published:** 2018-08-31

**Authors:** Trevor Gibbs

**Affiliations:** 1 AMEE

This review has been migrated. The reviewer awarded 5 stars out of 5 For many involved in the implementation of new educational programmes, the three-pronged approach usually suffices- " think about it, do it and evaluate it", but as the authors suggest, "..smooth implementation of high-stake endeavors such as developing and successfully launching educational innovations ranging from curricular sessions to new programs under time constraints" comes with careful consideration and within a logical structure.This carefully written paper provides some very useful practical tips for anyone involved in curricula programme activities. I enjoyed reading from it, learned a great amount and appreciated the informative list of references. The addition of the adaptive templates added to the overall value of the paper.I would suggest that this is an essential paper for those educators, administrators and secretariat staff involved in curriculum planning.

Reviewer Expertise:

NA

No decision status is available

# Reviewer report: Reviewer response for version 1

**DOI:** 10.21956/mep.19564.r29015

**Published:** 2018-05-24

**Authors:** Nandalal Gunaratne

**Affiliations:** 1 Faculty of Medicine, Wayamba University of Sri Lanka

This review has been migrated. The reviewer awarded 5 stars out of 5 This is a very useful and helpful set of tools for medical educators. I am most grateful for the time and effort the authors have put in and for allowing open access. The Excel based tool looks somewhat overwhelming. I will give feedback after I use it in my current education projects and future ones.

Reviewer Expertise:

NA

No decision status is available
